# *Drosophila Ref1*/*ALYREF* regulates transcription and toxicity associated with ALS/FTD disease etiologies

**DOI:** 10.1186/s40478-019-0710-x

**Published:** 2019-04-29

**Authors:** Amit Berson, Lindsey D. Goodman, Ashley N. Sartoris, Charlton G. Otte, James A. Aykit, Virginia M.-Y. Lee, John Q. Trojanowski, Nancy M. Bonini

**Affiliations:** 1 Department of Biology, University of Pennsylvania, Philadelphia, PA 19104 USA; 2 Neuroscience Graduate Group, Perelman School of Medicine, University of Pennsylvania, Philadelphia, PA 19104 USA; 3 Department of Pathology and Laboratory Medicine, Perelman School of Medicine, University of Pennsylvania, Philadelphia, PA 19104 USA

**Keywords:** *ALYREF*, *Ref1*, *Drosophila*, Amyotrophic lateral sclerosis (ALS) (Lou Gehrig disease), Ataxin-2, *C9orf72*, mRNA, Neurodegeneration, Nuclear transport, TAR DNA-binding protein 43 (TDP-43) (*TARDBP*)

## Abstract

RNA-binding proteins (RBPs) are associated with amyotrophic lateral sclerosis (ALS) and frontotemporal dementia (FTD), but the underlying disease mechanisms remain unclear. In an unbiased screen in *Drosophila* for RBPs that genetically interact with TDP-43, we found that downregulation of the mRNA export factor *Ref1* (fly orthologue to human *ALYREF*) mitigated TDP-43 induced toxicity. Further, *Ref1* depletion also reduced toxicity caused by expression of the *C9orf72* GGGGCC repeat expansion. *Ref1* knockdown lowered the mRNA levels for these related disease genes and reduced the encoded proteins with no effect on a wild-type Tau disease transgene or a control transgene. Interestingly, expression of TDP-43 or the GGGGCC repeat expansion increased endogenous *Ref1* mRNA levels in the fly brain. Further, the human orthologue ALYREF was upregulated by immunohistochemistry in ALS motor neurons, with the strongest upregulation occurring in ALS cases harboring the GGGGCC expansion in *C9orf72*. These data support *ALYREF* as a contributor to ALS/FTD and highlight its downregulation as a potential therapeutic target that may affect co-existing disease etiologies.

**Electronic supplementary material:**

The online version of this article (10.1186/s40478-019-0710-x) contains supplementary material, which is available to authorized users.

## Introduction

Amyotrophic lateral sclerosis (ALS) and frontotemporal dementia (FTD) are neurodegenerative diseases that share clinical, genetic, and pathologic hallmarks [50]. In recent years, mutations in a number of RNA binding proteins (RBPs) have been discovered in ALS/FTD, highlighting RNA-centric mechanisms in disease [62]. Of note, TDP-43 (encoded by the *TARDBP* gene) was identified as the primary component of ubiquitinated inclusions in ALS cases and a subset of FTD cases [40]. As an RBP, TDP-43 has been found to mediate a number of pathways related to RNA metabolism [28].

Recent investigations have revealed factors that may mediate TDP-43 associated ALS/FTD. An intermediate length polyglutamine repeat expansion (PolyQ) within another RBP, Ataxin-2*,* was defined as a risk factor for ALS that enhances the toxicity of TDP-43 [15]. Further, in genetic cases of familial ALS and FTD with TDP-43 pathology (FTD-TDP), a GGGGCC hexanucleotide repeat expansion (termed G4C2) in *C9orf72* was identified as the most common mutation [13, 42]. Interestingly, mice expressing expanded G4C2 have TDP-43 pathology [7]. Links between TDP-43 and G4C2 may converge on RNA metabolism as G4C2 expansions contribute to disruptions in various aspects of RNA processes [2, 27, 59, 61]. Altogether, accumulating studies strongly point to altered RNA biology as a critical component of disease etiology in ALS and FTD [9, 21].

Here, using a directed screen aimed at RNA-interacting proteins in *Drosophila*, we identified that *RNA and export factor binding protein 1* (*Ref1*), an orthologue of human *ALYREF* (also known as *THOC4*), modulates toxicity associated with TDP-43, TDP-43 with Ataxin-2, and G4C2. ALYREF is a component of the TRanscription and EXport (TREX) complex, a conserved complex that links transcription and processing of mRNAs to their export from the nucleus into the cytoplasm [20, 23, 48, 63]. Impaired nucleocytoplasmic shuttling has been identified as a major pathway impacted in C9+ ALS/FTD [8, 16, 43, 60]. Evidence also suggests that ALYREF may play a more direct role in transcription beyond mRNA export [5, 41, 49, 52, 58].

Using *Drosophila,* we show that knockdown of *Ref1* suppresses the toxicity of multiple related ALS/FTD genes (TDP-43, TDP-43 with Ataxin-2, and G4C2), providing the first evidence that it can mediate TDP-43-associated toxicity independent of G4C2 [16]. Further, depletion of *Ref1* using RNAi caused reductions in mRNA level and concomitant reduction in disease protein levels produced from TDP-43 and G4C2 disease genes, but not from a Tau disease gene. Interestingly, endogenous *Ref1* mRNA became upregulated in the TDP-43 and G4C2 fly models and ALYREF protein (the human *Ref1* orthologue) is upregulated by immunohistochemistry in ALS motor neurons. Upregulation is strongest in C9+ ALS cases, which harbor the G4C2 expansion and, presumably, have TDP-43 pathology [1, 13, 38, 42], compared to C9- ALS cases, which are expected to have only TDP-43 pathology [14, 32]. These data argue that a feed-forward loop may exist between the expression of *ALYREF* and disease genes, while highlighting *ALYREF* as an important disease modifier that may represent a therapeutic target of multiple co-existing disease etiologies.

## Results

### RNAi screen identifies *Ref1* as a strong suppressor of TDP-43 associated toxicity

**Fig. 1:**
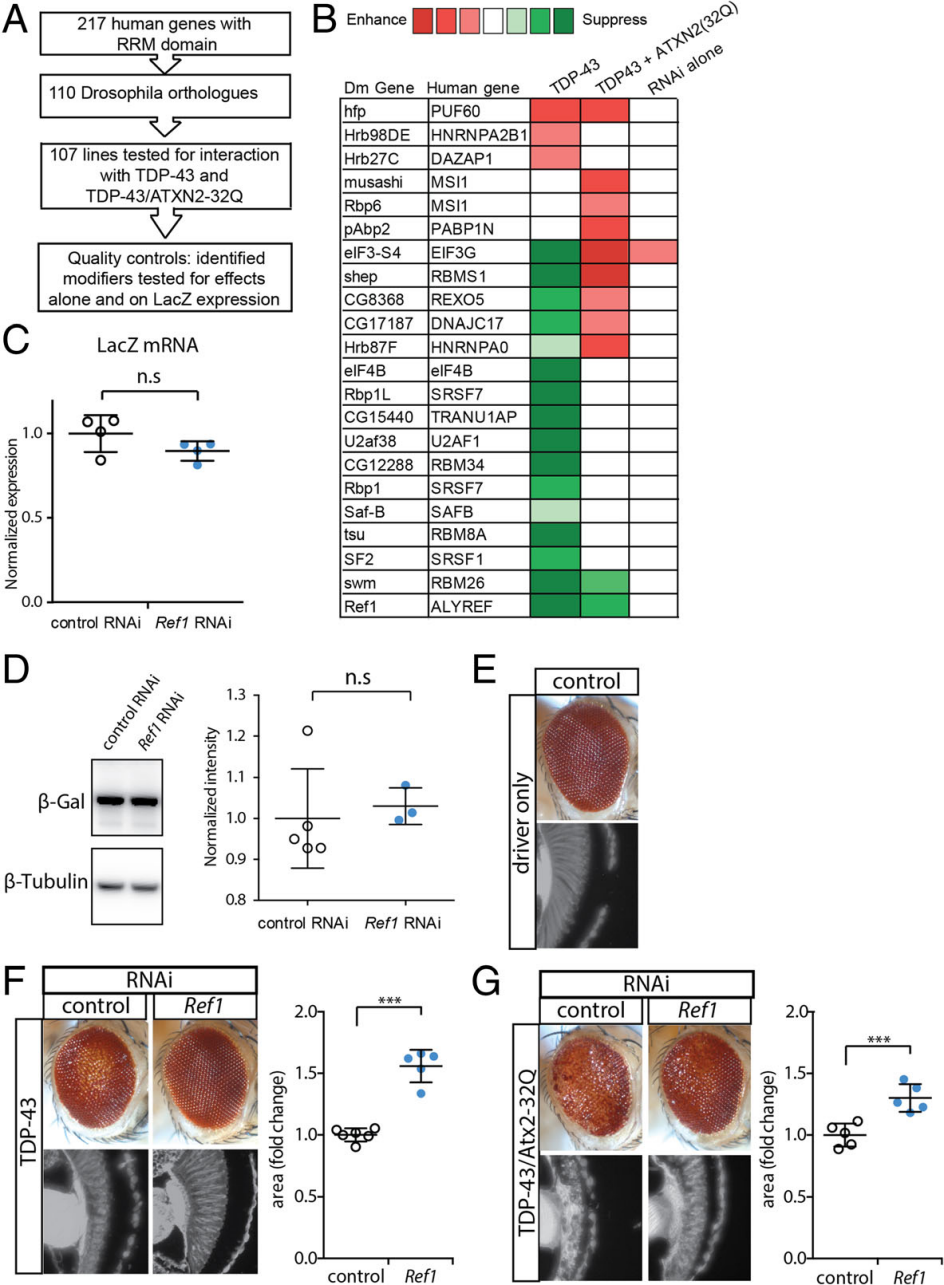
An in vivo RNAi screen identified *Ref1* among RNA-binding protein genes as a modifier of TDP-43 and ATXN2-32Q toxicity. **a** Screen overview. **b** Heatmap of modifiers identified in the screen. Enhancers in shades of red and suppressors in green. **c** *Ref1* knockdown does not affect the mRNA level of a reporter LacZ transgene. n.s. not statistically significant, two-tailed Student’s t-test. **d** *Ref1* knockdown does not affect the β-galactosidase protein levels produced from a reporter LacZ transgene. n.s. not statistically significant, two-tailed Student’s t-test. Genotypes for (**c**) and (**d**). Control RNAi: *GMR-GAL4, UAS-LacZ/+; UAS-control RNAi (JF01355)/+*. Ref1 RNAi: *GMR-GAL4, UAS-LacZ/+; UAS-Ref1 RNAi (HMS01301)/+*. **e** External image (top) and cross section (bottom) of the fly eye. Genotype: *YH3-GAL4/UAS-control RNAi (JF01355)*. **f** *Ref1* RNAi suppresses TDP-43-mediated neurodegeneration. Genotypes. Control: *UAS-TDP-43/+; YH3-GAL4/UAS-control RNAi (JF01355)*. Ref1: *UAS-TDP-43/+; YH3-GAL4/UAS-Ref1 RNAi (HMS01301)*. Quantification of retinal area from *Drosophila* head sections shown on the right. ***p < 0.001, two-tailed Student’s t-test. **g** *Ref1* RNAi suppresses TDP-43+ ATXN2(32Q)-mediated neurodegeneration. Genotypes. Control: *UAS-TDP-43, UAS-ATXN2-32Q/+; YH3-GAL4/UAS-control RNAi (JF01355)*. Ref1: *UAS-TDP-43, UAS-ATXN2-32Q /+; YH3-GAL4/UAS-Ref1 RNAi (HMS01301)*. Quantification of retinal area shown on the right. ***p < 0.001 two-tailed Student’s t-test. For all graphs, individual data points are shown with mean +/− standard deviation

We established an in vivo RNAi screen in *Drosophila melanogaster* to identify RNA interacting proteins that can modify the toxicity of ALS-associated genes (Fig. 1a). The screen was performed against two *Drosophila* models of TDP-43 proteinopathy. Human TDP-43 was expressed with or without added *ATXN2* bearing an intermediate polyglutamine repeat expansion of 32 glutamines (Q), an established risk factor for ALS [15, 39]. The disease-associated genes, as well as the RNAi transgenes, were expressed in the fly eye using the *GAL4/UAS* system [4]. 107 *Drosophila* genes that encode proteins with an RNA recognition motif (RRM) and have a direct human orthologue were targeted by RNAi. We focused on RRM containing genes because many of the genes associated with ALS contain such domains [21].

The screen identified a total of 22 modifiers (Fig. 1b, full screen results described in Additional file 1: Table S1). Of these, 3 showed strong and consistent effects between the TDP-43 and TDP-43/ATXN2-32Q models: *Half pint* (*hfp*, also known as *pUF68*) RNAi enhanced TDP-43 and TDP-43/ATXN2-32Q toxicity, while *Ref1* and *second mitotic wave missing* (*swm*) RNAi suppressed the degenerative eye effect. We used a reporter gene, *LacZ*, to rule out whether these modifiers affected the *GAL4/UAS* expression system of the transgenes. This showed that RNAi to *hfp* increased and *swm* decreased the levels of β-galactosidase protein (Additional file 2: Figure S1A); importantly, *Ref1* had no effect on the RNA or protein expression from the control LacZ gene (Fig. 1c, d). We confirmed efficient knockdown of *Ref1* by the RNAi line (Additional file 2: Figure S1B). *Ref1* was the strongest hit from the screen as it suppressed neurodegeneration caused by TDP-43 (Fig. 1c, d) and TDP-43/ATXN2-32Q (Fig. 1e), without an effect on expression of an unrelated control protein.

### *Ref1* knockdown suppresses TDP-43 toxicity by reducing its expression

**Fig. 2:**
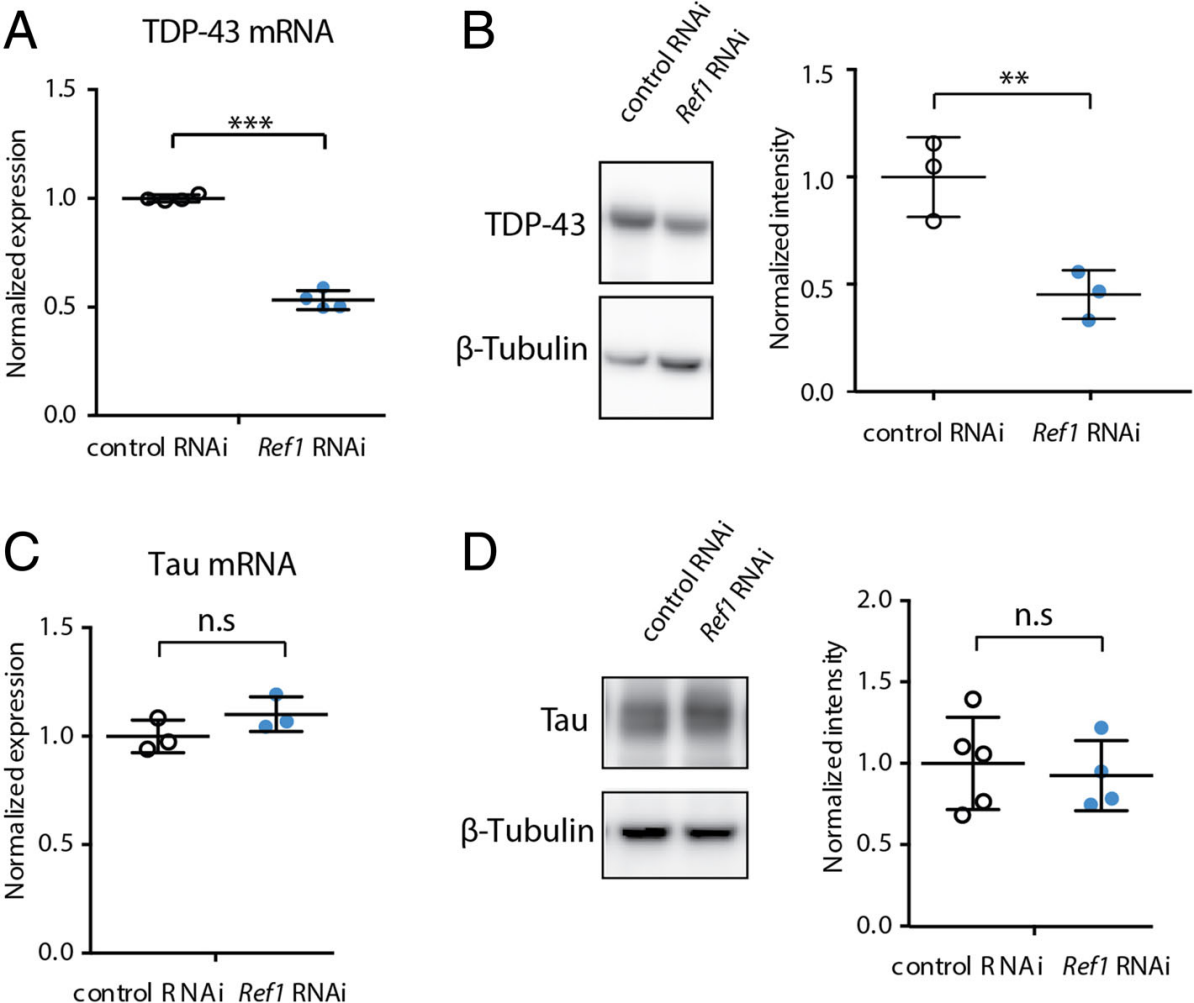
*Ref1* reduction lowers TDP-43 RNA and protein abundance. **a** *Ref1* Knockdown reduces TDP-43 mRNA levels in *Drosophila* heads. ***p < 0.001 two-tailed Student’s t-test. **b** *Ref1* knockdown reduces TDP-43 protein levels in *Drosophila* heads. Western immunoblot analysis of human TDP-43 and β-Tubulin as loading control. *p < 0.05 two-tailed Student’s t-test. Genotypes for (**a**) and (**b**). Control RNAi: *UAS-TDP-43/+; YH3-GAL4/UAS-control RNAi (JF01355)*. Ref1: *UAS-TDP-43/+; YH3-GAL4/UAS-Ref1 RNAi (HMS01301)*. **c** Loss of *Ref1* does not alter Tau mRNA levels. n.s. not statistically significant, two-tailed Student’s t-test. **d** Loss of *Ref1* does not alter Tau protein levels. n.s not statistically significant, two-tailed Student’s t-test. Genotypes for (**c**) and (**d**). Control RNAi: *YH3, UAS-Tau/ UAS-control RNAi (JF01355)*. *Ref1* RNAi: *YH3, UAS-Tau/ UAS-Ref1 RNAi (HMS01301).* For all graphs, individual data points are shown with mean +/− standard deviation

As ALYREF is a known mediator of RNA metabolism, we hypothesized that loss of *Ref1* could alter the level of expression of the TDP-43 mRNA. Total RNA was extracted from fly heads co-expressing TDP-43. TDP-43 mRNA levels were determined using quantitative realtime polymerase chain reaction (qRT-PCR). Knockdown of *Ref1* significantly reduced TDP-43 mRNA levels (Fig. 2a). Western immunoblot analysis was then used to determine the effects of *Ref1* knockdown on TDP-43 protein levels. Consistent with its effects on TDP-43 mRNA, *Ref1* RNAi reduced TDP-43 protein levels (Fig. 2b).

To gain an understanding if the effect of *Ref1* knockdown was universal to all disease genes, we tested for effects of *Ref1* depletion on expression of the disease gene Tau. Tau (encoded by the *MAPT* gene) is hyperphosphorylated and aggregates in a class of neurodegenerative diseases termed Tauopathies, which includes a subset of FTD cases lacking abnormal TDP-43 inclusions [47]. Thus, Tau represents an unrelated disease etiology as it does not co-occur with TDP-43 pathology. The reduced expression from the TDP-43 transgene in response to *Ref1* RNAi was specific to this disease gene as *Ref1* RNAi had no effect on mRNA expression (Fig. 2c) or protein expression (Fig. 2d) from a wild-type Tau transgene. These data indicated that *Ref1* mitigated toxicity by selectively reducing levels of the disease TDP-43 mRNA.

### *Ref1* knockdown reduced expression from a G4C2 transgene, resulting in reduced G4C2-toxicity

**Fig. 3:**
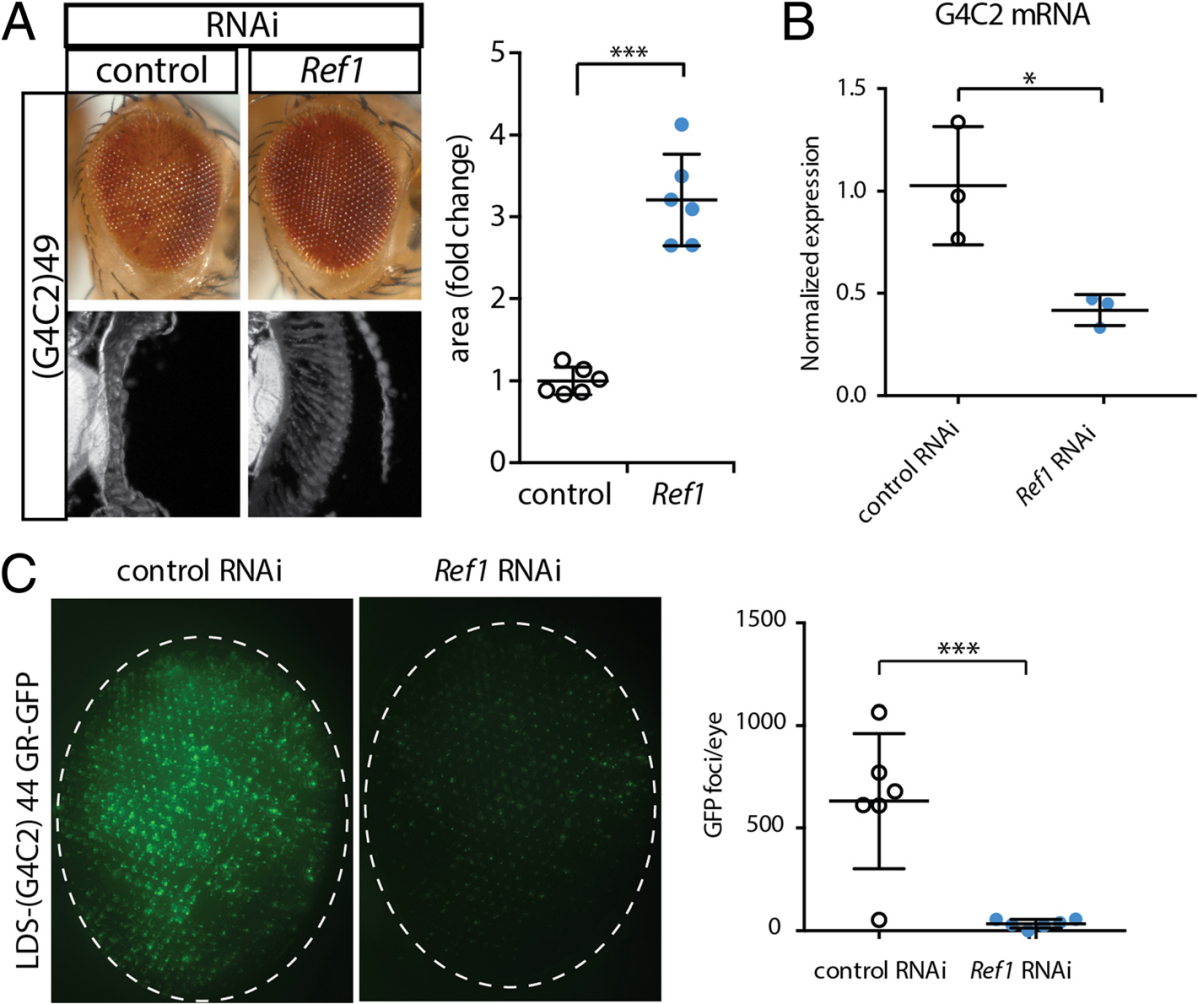
*Ref1* reduction lowers G4C2 RNA and dipeptide abundance. **a** *Ref1* RNAi suppresses G4C2 repeat-mediated neurodegeneration. Genotypes. Control: *UAS-(G4C2)49, YH3-GAL4/UAS-control RNAi (JF01355)*. Ref1: *UAS-(G4C2)49, YH3-GAL4/UAS-Ref1 RNAi (HMS01301)*. Quantification of retinal area shown on the right. ***p < 0.001, two-tailed Student’s t-test. **b** *Ref1* knockdown reduces G4C2 mRNA levels in *Drosophila* heads. **d** *Ref1* knockdown reduces GR-GFP protein levels in the *Drosophila* eye. **e** Quantification of GR-GFP signal. Genotypes for (**b**) and (**c**). Control RNAi: *(G4C2)44 [GR-GFP], YH3/ UAS-control RNAi (JF01355)*. *Ref1* RNAi: *(G4C2)44 [GR-GFP], YH3/ UAS-Ref1 RNAi (HMS01301)*. ***p < 0.001 two-tailed Student’s t-test. For all graphs, individual data points are shown with mean +/− standard deviation

ALYREF (human *Ref1* protein homolog) was previously reported to bind G4C2 RNA [11, 19] and the presence of G4C2 expansions is found in TDP-43-associated ALS/FTD. Thus, we hypothesized that *Ref1* may be able to also modify toxicity caused by expression of > 30 G4C2 repeats. *Ref1* had previously been proposed as a modifier of G4C2-induced toxicity, causing accumulation of total mRNA within the nucleus when depleted [16]. However, *Ref1* downregulation was not able to significantly modify toxicity associated with a shorter (G4C2)36 gene [19]. To further examine *Ref1* as a potential modifier of expanded G4C2, we used a fly model expressing (G4C2)49 that induces neurodegeneration [6, 18, 26, 37]. Co-expression of *Ref1* RNAi and expanded G4C2 resulted in suppressed degeneration in the fly eye (Fig. 3a). Importantly, this effect was consistent in both the external eye – seen by reduced pigment loss and recovered ommatidial organization – and in the internal retina tissue – seen by increased tissue integrity.

Given the effect of *Ref1* RNAi on expression of the TDP-43 mRNA (see Fig. 2) and that recent evidence supports that ALYREF may play a more global role in transcription [5, 41, 49, 52, 58], we hypothesized that *Ref1* may also alter expression of expanded G4C2. To assess the effects of *Ref1* on expression, total RNA was extracted from fly heads co-expressing the G4C2 repeat transgene together with RNAi to *Ref1* or a control. G4C2 RNA levels were determined using qRT-PCR [18]. Knockdown of *Ref1* caused a significant reduction in the mRNA levels of G4C2 repeats (Fig. 3b).

G4C2 RNA can produce three dipeptide repeats (DPR) by non-AUG (RAN-) translation: glycine-arginine (GR), glycine-alanine (GA), glycine-proline (GP) [1, 34]. These DPR can form aggregates in ALS/FTD tissue. Of these, GR dipeptides have been consistently shown to be toxic in *Drosophila* with GA showing mild effects, and GP no toxicity [16, 36]. To determine if *Ref1* knockdown effected GR protein levels, we utilized a *Drosophila* transgenic line that expresses a G4C2 repeat with a green fluorescent protein (GFP) tag in frame with the GR dipeptide [18]. Fluorescent imaging revealed that knockdown of *Ref1* dramatically reduced GR-GFP accumulation (Fig. 3c). Blinded quantification of the GFP signal revealed a significant and consistent downregulation of the GR-GFP signal.

Overall, modification of G4C2-toxicity was consistent with a previous report [16], while our data further show that *Ref1* loss alters the mRNA level of G4C2. Importantly, as *Ref1* also altered expression of TDP-43 mRNA, it may serve as a unique target that can modify these two co-existing pathologies simultaneously.

### *Ref1* is upregulated in response to TDP-43 or G4C2 expression in the adult fly brain

**Fig. 4:**
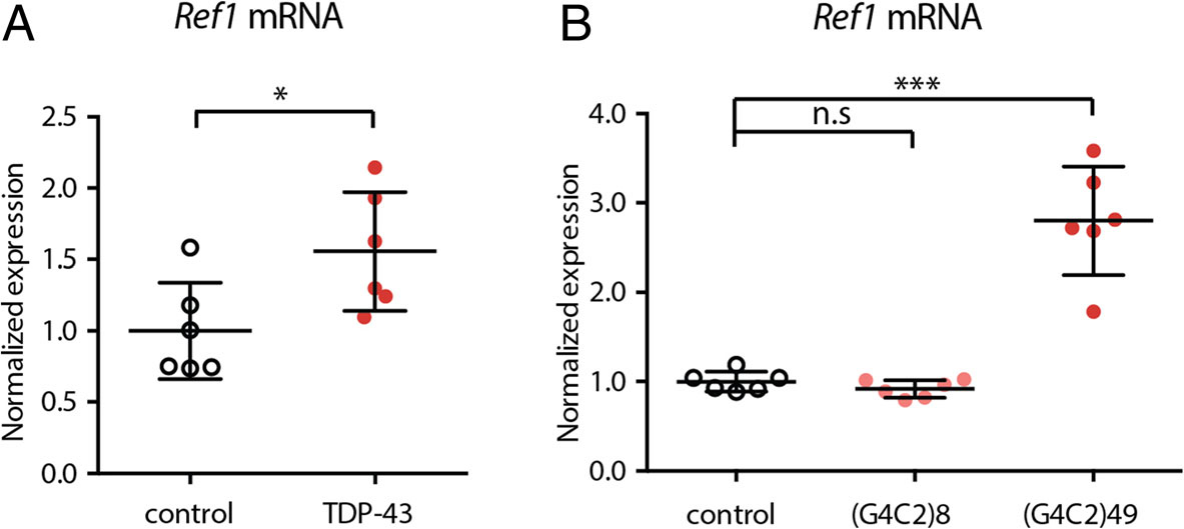
*Ref1* mRNA level is increased in *Drosophila* ALS/FTD models. **a** *Ref1* mRNA levels in *Drosophila* heads are increased by TDP-43 expression. *p < 0.05 two tailed Student’s t-test. Genotypes. Control: *elavGS/+*. TDP-43: *elavGS/UAS-TDP-43*. **b** *Ref1* mRNA levels in *Drosophila* heads are increased by long (49), but not short (8) G4C2 repeats expression. One-way ANOVA with Dunnett’s multiple comparisons test, F(2,15) = 52.09, *p* < 0.001. ***p < 0.001, n.s not statistically significant. Genotypes. Control: *elavGS/+.* (G4C2)8: *elavGS/UAS-(G4C2)8*. (G4C2)49: *elavGS/UAS-(G4C2)49*. For all graphs, individual data points are shown with mean +/− standard deviation

As our data supported that knockdown of *Ref1* modulates toxicity of TDP-43 and G4C2, we considered whether expression of endogenous *Ref1* might be impacted by expression of these disease genes. To examine this, we expressed TDP-43 or a G4C2 repeat expansion in neurons of adult flies for 16d using the drug-inducible neuronal driver ElavGS, and then measured endogenous *Ref1* mRNA levels in fly heads by qRT-PCR. Interestingly, endogenous *Ref1* mRNA levels were significantly increased in animals expressing TDP-43 (Fig. 4a). Further, *Ref1* mRNA levels were significantly increased upon expression of long and toxic (49 units), but not short and inert (8 units), G4C2 repeats (Fig. 4b). Taken together, these data suggest that *Ref1* activity is upregulated by TDP-43 and toxic expanded G4C2, and that reduction of that activity by RNAi in *Drosophila* is beneficial.

### ALYREF protein levels are increased in human ALS motor neurons

Thus far, we found that *Ref1* depletion in flies could suppress toxicity caused by expression of ALS/FTD disease genes TDP-43 and G4C2. Suppression was the result of reduced mRNA levels of these disease genes. Moreover, expression of TDP-43 and expanded G4C2 in the adult fly nervous system caused upregulation of endogenous *Ref1*. Given these findings, we were curious as to whether there may be an alteration in the expression of *ALYREF*, the human *Ref1* orthologue, in human disease.

**Fig. 5:**
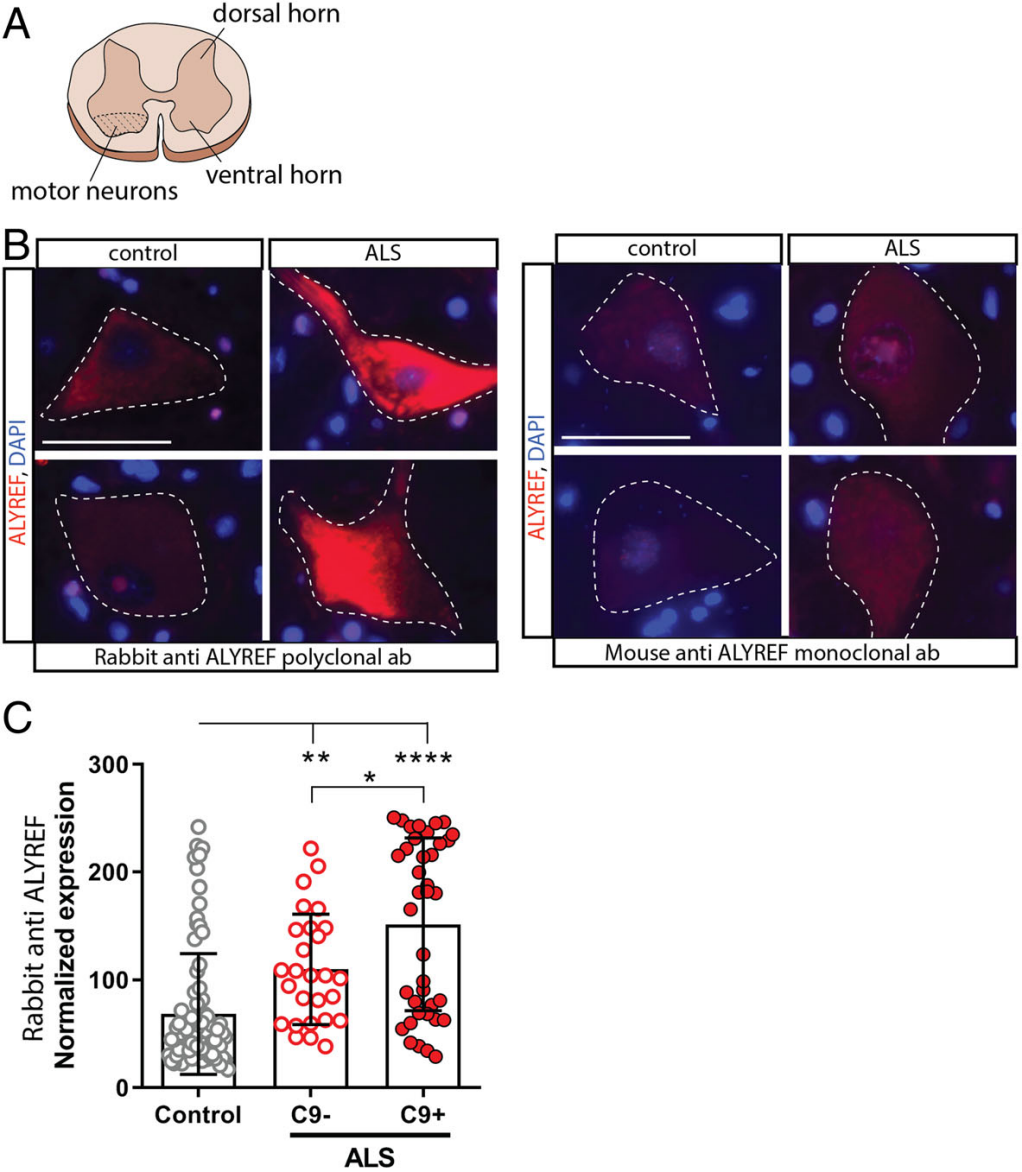
ALYREF protein levels are increased in human ALS patient motor neurons. **a** Schematic depicting the location of spinal cord motor neurons imaged in (**b**). **b** Fluorescent immunostaining of ALYREF in lumbar spinal cord sections from control and ALS cases. 2 independent ALYREF antibodies were used. Scale bar 50 μm. **c** Image intensity quantification using the rabbit polyclonal antibody targeting ALYREF protein. Control *n* = 83 cells from *n* = 9 cases. C9- ALS *n* = 27 cells from *n* = 3 cases. C9+ ALS *n* = 38 cells from *n* = 3 cases. ****p < 0.0001, **p < 0.01, *p < 0.05 One-way ANOVA, Tukey’s multiple comparisons test, F(2,145) = 23.87. For all graphs, individual data points are shown with mean +/− standard deviation

Immunofluorescence imaging for ALYREF protein was performed on lumbar spinal cord tissue sections to define any changes in localization and/or expression of ALYREF in disease (Fig. 5a). ALYREF is known to predominantly localize to the nucleus, although it can translocate to the cytoplasm as it accompanies exported nuclear mRNAs into the cytoplasm [3, 54, 57, 63]. Using two independent antibodies to ALYREF, we found that ALYREF localized to both the nucleus and cytoplasm in ALS motor neurons (Fig. 5b; patient information can be found in Additional file 3: Table S2). Importantly, ALYREF protein levels were significantly upregulated in ALS motor neurons compared to controls. Blinded quantification of ALYREF intensity revealed that upregulation of ALYREF was stronger in C9+ ALS cases, which have both TDP-43 pathology and the G4C2 repeat expansion present in *C9orf72* [1, 13, 38, 42], compared to C9- ALS cases, which have only TDP-43 pathology [14, 32] (Fig. 5c). These data are consistent with the fly data showing that *Ref1* (the orthologue of ALYREF in *Drosophila*) is upregulated upon expression of TDP-43 and G4C2. Moreover, these data support ALYREF dysregulation in human ALS/FTD.

## Discussion

Herein, we identified that knockdown of *Ref1* is a suppressor of toxicity of TDP-43 and TDP-43 co-expressed with Ataxin-2 in a fly-based, modifier screen of 107 RNA binding proteins containing RNA recognition motifs (RRMs). Suppression of TDP-43 toxicity was associated with downregulation of TDP-43 on both the RNA and protein levels in *Ref1*-depleted animals. Neither RNA nor protein expression from a control LacZ gene or an unrelated disease gene, Tau, were altered upon *Ref1* RNAi. Additional investigation revealed that *Ref1* depletion also suppressed toxicity of a related ALS/FTD mutation: the expanded G4C2 repeat (also [16]). Significant reductions in G4C2 RNA and concomitant reductions in toxic GR-dipeptide were seen upon *Ref1* downregulation in G4C2 expressing animals. Intriguingly, expression of TDP-43 or expanded (G4C2)49 disease transgenes within the adult fly nervous system were associated with an upregulation of endogenous *Ref1* RNA, suggesting a feed-forward mechanism may be occurring. Consistent with these results, previous work on TDP-43 expressing human cells also reported ALYREF upregulation in the cytoplasmic fraction [35]. Importantly, our data indicate that ALYREF is upregulated at the protein level in motor neurons of ALS patients, with patients bearing the expanded G4C2 repeat mutation showing significantly higher ALYREF protein levels (see Fig. 5).

*ALYREF* is involved in several related pathways that may lead to suppression of TDP-43 and G4C2 toxicity when depleted. As a member of the TREX complex, ALYREF is best known for its function in mediating nucleocytoplasmic transport of mRNAs [20, 23, 48, 63]. Within this complex, ALYREF serves as an adaptor protein between mRNA and export factors NXF1/p15. Importantly, disruptions in nucleocytoplasmic transport may be a mechanism of both TDP-43- and G4C2-associated disease [8, 16, 43]. In contrast to its role in transport, ALYREF has also been reported to mediate RNAPII-driven transcription because it interacts with a number of transcription factors and depletion of ALYREF can reduce RNAPII occupancy for a subset of genes [20, 23, 49]. Our findings further highlight transcription as an additional mechanism underlying the role of *Ref1*/*ALYREF* in disease, as we see downregulation of the TDP-43 and G4C2 repeat transgene mRNA levels (see Figs. 2, 3).

Interestingly, ALYREF can bind both sense G4C2 and antisense G2C4 RNA [10, 11, 19, 30], suggesting that an interaction between ALYREF/Ref1 and G4C2 can be direct. For TDP-43, we present the first evidence that ALYREF also modulates TDP-43-associated toxicity. Further investigation into whether TDP-43 RNA interacts with ALYREF protein are needed to determine whether this may also be by direct binding. Overall, our data suggest that there are commonalities in the ability of ALYREF to modify expression of these two disease genes over a non-disease transcript. While it is clear that ALYREF is selective to specific transcripts [17, 24, 33, 49], what defines an ALYREF interacting gene is currently unknown. Only recently have there been studies that shed light on underlying mechanisms, defining ALYREF as an m^5^C reader [57] and potential ALYREF binding motifs [46].

*ALYREF* may serve as a unique therapeutic target in ALS as its depletion was able to suppress both TDP-43- and G4C2-induced toxicity. Further investigations into the role of *ALYREF* in global transcription, global mRNA export, and effects on disease-associated pathways are needed to define it as a potential therapeutic target [24, 53, 55]. Previous work showed that ALYREF is not essential for bulk mRNA export from the nucleus in *Drosophila* and *C. elegans* [17, 33] and only a subset of mRNAs are affected when ALYREF is depleted in human cells [41, 49]. Importantly, our data indicate that ALYREF is upregulated in motor neurons of ALS patients, with patients bearing the expanded G4C2 repeat mutation showing significantly higher ALYREF levels (see Fig. 5). Overall, these data support previous findings that there may be overlapping mechanisms underlying these related disease etiologies [7, 12, 29, 44]. Interesting to C9+ disease, ALYREF has been reported to interact with Iws1 – a transcription factor that binds SPT4/5 RNAPII-elongation factors [31, 58]. SPT4/5 has recently been identified as unique transcriptional regulators of expanded G4C2 [26], suggesting that ALYREF is positioned to be a protein that may couple G4C2 transcription to nuclear export machinery. TREX proteins (including ALYREF) were also found to interact with Matrin 3 [3], another RBP that is mutated in ALS [22], suggesting that ALYREF may play a role in multiple types of ALS/FTD.

Despite recent advances in our understanding of the molecular mechanisms underlying ALS/FTD, there is an urgent and unmet need to develop effective therapeutics. Our results identify ALYREF as a potential novel target that is increased in ALS motor neurons, and whose downregulation may suppress the toxicity of multiple ALS and FTD associated genes.

## Material and methods

### Drosophila stocks and crosses

Flies were grown on standard cornmeal molasses agar with dry yeast. Stock lines were maintained at 18 °C. Transgenic lines used in this study were: *UAS-TDP-43/CyO; GMR-GAL4 (YH3)/TM6B*. *UAS-TDP-43(37 M), UAS-hATXN2-32Q (F26)/CyO; GMR-GAL4 (YH3)/TM6B* and *GMR-GAL4 (YH3)/TM3, Sb* [25]. *UAS-(G4C2)49, GMR-GAL4 (YH3)/TM6, Sb* [18, 26, 37]. *UAS-LDS-(G4C2)4,42,44[GR-GFP*] [18]. *UAS-Tau*^*WT*^ [56]. RNAi lines from the Transgenic RNAi Project (TRiP) and mutant lines were obtained from the Bloomington *Drosophila* Stock Center. Additional RNAi lines were obtained from the Vienna *Drosophila* Resource Center. Complete list of RNAi lines used in this study is found in Additional file 4: Table S3.

For the genetic screen, virgin female flies were selected from each disease model line or from driver-only lines and were crossed to males harboring RNAi transgenes at 25 °C, under normal light/dark cycles. Male progeny of the appropriate genotypes were selected, aged to 3-5d at 25 °C. For external eye imaging, flies were anesthetized with ether for 10 min, placed on a glass slide and imaged with Leica Z16 APO. For internal eye morphology, male flies from the same crosses were fixed in Bouin’s solution (Sigma-Aldrich) for 120 h, embedded in paraffin, sectioned on a Leica RM2255 microtome and mounted on SuperFrost Plus slides (Fischer Scientific). Slides were dried overnight at room temperature, baked for 1 h at 56 °C, and paraffin was removed with Histoclear (National Diagnostics). Slides were mounted with coverslips using Cytoseal XYL (Thermo Scientific) and imaged using an upright Leica fluorescent microscope.

### Western immunoblots

20 fly heads were homogenized in 50 μl LDS sample buffer (Invitrogen) including 5% beta-mercaptoethanol (Sigma-Aldrich). Samples were boiled at 95 °C for 5 min and centrifuged at 15,000 g for 5 min at 4 °C. The supernatant was collected and stored at − 20 °C until loaded on 4–12% Bis-Tris NuPAGE gels (Invitrogen) using 5 μl of sample per well. Gel electrophoresis was performed at 140 V for 70 min and the gels were blotted on a PVDF membrane using XCell II (Invitrogen) at 30 V for 1 h. Membranes were blocked in 3% bovine serum albumin in tris buffered saline with 0.1% Tween20 (TBST) for 30 min and incubated with primary antibodies in blocking buffer over-night at 4 °C. Following washes in TBST, membranes were incubated with HRP-conjugated secondary antibodies (Jackson Immunoresearch) at 1:10,000 for 2 h, washed and the luminescent signal was developed using ECL prime (Amersham) and detected with Amersham Imager 600. Primary antibodies: anti β-tubulin (CAT#E7, DSHB, 1:500), anti β-Galactosidase (CAT#Z378A, Promega, 1:2000), anti-TDP-43 (CAT#10782, Proteintech, 1:1000), anti Tau (CAT#A0024, Dako, 1:1000).

### Real-time PCR

RNA was extracted using Trizol Reagent (ThermoFisher Scientific), according to the manufacturer’s instructions. RNA concentration was determined using Nanodrop (Nanodrop) and RNA quality was assessed using 1% agarose gel-electrophoresis. 400 ng RNA was used per reverse-transcription reaction using the High Capacity cDNA Reverse Transcription Kit (Applied Biosystems) in a 20 μl total reaction volume using random primers. cDNA was then used as template for real-time qPCR using Fast SYBR Green Master Mix (Applied Biosystems). Real-time PCR was performed on the Applied Biosystems ViiA7 machine using 384-well format in technical duplicates. For each primer set, a serial dilution curve validated primer efficiency. Melting curve analysis confirmed the existence of one amplicon. Primers were designed using Primer3 (http://bioinfo.ut.ee/primer3-0.4.0/primer3/).

Primer sequences are listed in Additional file 5: Table S4.

### Human tissue – immunofluorescence

For immuno-fluorescence, Superfrost slides (Fisher Scientific) with paraffin sections were deparaffinized in xylene, 100, 90 and 70% ethanol. Following a brief rinse in water, antigen retrieval was performed by boiling the samples for 10 min in citric buffer pH 6 (10 mM citric acid, pH 6). Slides were cooled to room temperature, rinsed with water and incubated in blocking buffer for 20 min (Tris buffered saline (TBS) with 2% bovine serum). Sections were circled with a liquid blocker PAP pen (Daido Sangyo) and primary antibodies in blocking buffer were incubated overnight at 4 °C. Following 3 washes in TBS, slides were blocked for 5 min and incubated with secondary antibodies for 2 h at room-temperature. Slides were washed 3 times in TBS. To quench autofluorescence, slides were washed for 1 min in 70% ethanol, incubated for 1 min in Sudan Black (0.3% in 70% ethanol), and washed 3 times in 70% ethanol. Final TBS wash was followed by 1 μg/ml DAPI to stain DNA, slides were rinsed in water, mounted with anti-fade mounting media (20 mM Tris pH 8.0, 0.5% N-propyl gallate, 80% glycerol), and sealed with clear nail polish. Quantification of fluorescent signal was performed with Fiji [45]. Imaging and quantification were performed blinded to disease status. Primary antibodies were used at 1:200 dilution: anti-ALYREF (mouse monoclonal, CAT# ab6141, abcam), anti-ALYREF (rabbit polyclonal, CAT# ab202894, abcam). Secondary antibodies were used at 1:200 dilution: anti-Rabbit IgG Alexa Fluor 594 (#A-11012, 1:200, Invitrogen). Details of human samples are described in Additional file 3: Table S2. Donor spinal cord samples following neuropathological evaluation were selected from the brain bank at the Center for Neurodegenerative Disease Research at the University of Pennsylvania [51]. Phosphorylated TDP-43 deposits were evaluated using the pS409/410 antibody (mAb, 1:500) [40]. Controls were defined as subjects who were cognitively normal and did not meet the threshold for a neurodegenerative or vascular dementia diagnosis during the neuropathological examination. Informed consent for autopsy was obtained for all patients from their next of kin.

### Statistical analysis

Statistical analysis was performed using Prism (Version 6, GraphPad). One-way ANOVA with Dunnett’s or Tukey’s multiple comparisons test or two tailed Student’s t-test were used as appropriate, with significance level set at 0.05.

## Additional files

Additional file 1: **Table S1.** RBP screen details. (PDF 162 kb)

Additional file 2: **Figure S1.** Extended data characterizing *Ref1* RNAi. **Figure S2.** Uncropped western blot images. (DOCX 977 kb)

Additional file 3: **Table S2.** Patient information. (PDF 56 kb)

Additional file 4: **Table S3**. *Drosophila* RNAi lines. (PDF 98 kb)

Additional file 5: **Table S4.** Primer sequences. (PDF 57 kb)

## Abbreviations

ALS: Amyotrophic lateral sclerosis
DPR: Dipeptide repeats
FTD: Frontotemporal Dementia
G4C2: Expanded (GGGGCC)_30+_ mutation found within *C9orf72*
GFP: Green fluorescent protein
qRT-PCR: Quantitative realtime polymerase chain reaction
RAN-translation: Repeat Associated Non-AUG translation
RBP: RNA binding protein
Ref1: RNA and export factor binding protein 1; fly orthologue to human *ALYREF*
RNP: Ribonucleoprotein
RRM: RNA recognition motif
TDP-43: TAR DNA-binding protein 43
TREX: TRanscription and EXport
