# Supplementary material for: Drosophila Ref1/ALYREF regulates transcription and toxicity associated with ALS/FTD disease etiologies
Source: Acta Neuropathol Commun. 2019 Apr 29;7:65. doi: 10.1186/s40478-019-0710-x (PMC6487524; doi:10.1186/s40478-019-0710-x)
Supplement: Supplementary file 1 — Table S1. RBP screen details. (PDF 162 kb) [file 40478_2019_710_MOESM1_ESM.pdf]

Table S1

Dm Gene	CG #	Stock #	Genotype	Source	Effect on TDP43, hAtx2		Effect on TDP43		Effect by itself	
A2bp1	CG32062	32476	y[1] sc[*] v[1]; P{y[+t7.7] v[+t1.8]=TRiP.HMS00478}attP2	Bloomington	no effect	0	no effect	0	no effect	0
aret	CG31762	38983	y[1] v[1]; P{y[+t7.7] v[+t1.8]=TRiP.HMS01899}attP40	Bloomington	no effect	0	no effect	0	no effect	0
B52	CG10851	37519	y[1] sc[*] v[1]; P{y[+t7.7] v[+t1.8]=TRiP.HMS01661}attP40	Bloomington	no effect	0	no effect	0	no effect	0
barc	CG6049	42504	y[1] v[1]; P{y[+t7.7] v[+t1.8]=TRiP.HMJ02069}attP40	Bloomington	no effect	0	no effect	0	no effect	0
bol	CG4760	v21536	w[1118]; P{GD10525}v21536	VDRC	no effect	0	no effect	0	no effect	0
bru-2	CG43065	50631	y[1] sc[*] v[1]; P{y[+t7.7] v[+t1.8]=TRiP.HMC02998}attP2	Bloomington	no effect	0	no effect	0	no effect	0
bru-3	CG43744	43318	y[1] sc[*] v[1]; P{y[+t7.7] v[+t1.8]=TRiP.HMS02702}attP40	Bloomington	no effect	0	no effect	0	no effect	0
caz	CG3606	34839	y[1] sc[*] v[1]; P{y[+t7.7] v[+t1.8]=TRiP.HMS00156}attP2	Bloomington	no effect	0	no effect	0	no effect	0
cbp20	CG12357	42596	y[1] sc[*] v[1]; P{y[+t7.7] v[+t1.8]=TRiP.HMS02428}attP40	Bloomington	no effect	0	no effect	0	no effect	0
CG10466	CG10466	55263	y[1] sc[*] v[1]; P{y[+t7.7] v[+t1.8]=TRiP.HMC03950}attP40	Bloomington	no effect	0	no effect	0	no effect	0
CG10948	CG10948	55280	y[1] sc[*] v[1]; P{y[+t7.7] v[+t1.8]=TRiP.HMC03967}attP40	Bloomington	no effect	0	no effect	0	no effect	0
CG11266	CG11266	44431	y[1] sc[*] v[1]; P{y[+t7.7] v[+t1.8]=TRiP.GLC01382}attP2/TM3, Sb[1]	Bloomington	no effect	0	no effect	0	no effect	0
CG11454	CG11454	GLC01372	y[1] v[1]; P{y[+t7.7] v[+t1.8]=TRiP.GLC01372}attP40	TRiP center	no effect	0	no effect	0	no effect	0
CG12288	CG12288	56037	y[1] sc[*] v[1]; P{y[+t7.7] v[+t1.8]=TRiP.HMC04345}attP40	Bloomington	no effect	0	suppression	3	no effect	0
CG1316	CG1316	v23851	w[1118]; P{GD7942}v23851/TM3	VDRC	no effect	0	no effect	0	no effect	0
CG13298	CG13298	42873	y[1] sc[*] v[1]; P{y[+t7.7] v[+t1.8]=TRiP.HMS02566}attP40	Bloomington	enhancement	-3	enhancement	-2	an effect	-2
CG14641	CG14641	v38790	w[1118]; P{GD8388}v38790/TM3	VDRC	no effect	0	no effect	0	no effect	0
CG14718	CG14718	38331	y[1] sc[*] v[1]; P{y[+t7.7] v[+t1.8]=TRiP.HMS01798}attP2	Bloomington	no effect	0	no effect	0	no effect	0
CG15440	CG15440	57807	y[1] v[1]; P{y[+t7.7] v[+t1.8]=TRiP.HMJ21815}attP40	Bloomington	suppression	1	Suppression	3	no effect	0
CG17187	CG17187	55702	y[1] sc[*] v[1]; P{y[+t7.7] v[+t1.8]=TRiP.HMC03923}attP40	Bloomington	enhancement	-1	Suppression	2	no effect	0
CG18259	CG18259	34081	y[1] sc[*] v[1]; P{y[+t7.7] v[+t1.8]=TRiP.HMS01089}attP2/TM3, Sb[1]	Bloomington	no effect	0	no effect	0	no effect	0
CG2931	CG2931	v20946	w[1118]; P{GD9858}v20946	VDRC	no effect	0	no effect	0	no effect	0
CG3294	CG3294	27296	y[1] v[1]; P{y[+t7.7] v[+t1.8]=TRiP.JF02606}attP2	Bloomington	no effect	0	no effect	0	no effect	0
CG3335	CG3335	17737	w[1118]; PBac{w[+mC]=PB}CG3335[c05958]/TM6B, Tb[1]	Bloomington	no effect	0	no effect	0	no effect	0
CG33713	CG33713	55374	y[1] sc[*] v[1]; P{y[+t7.7] v[+t1.8]=TRiP.HMC04062}attP40/CyO	Bloomington	no effect	0	no effect	0	no effect	0
CG34354	CG34354	33674	y[1] sc[*] v[1]; P{y[+t7.7] v[+t1.8]=TRiP.HMS00538}attP2	Bloomington	no effect	0	no effect	0	no effect	0
CG34362	CG34362	v39749	w[1118]; P{GD7852}v39749	VDRC	no effect	0	no effect	1	no effect	0
CG4119	CG4119	v26395	w[1118]; P{GD11222}v26395	VDRC	no effect	0	no effect	0	no effect	0
CG42458	CG42458	42506	y[1] v[1]; P{y[+t7.7] v[+t1.8]=TRiP.HMJ02071}attP40	Bloomington	no effect	0	no effect	3	no effect	0
CG4266	CG4266	v26472	w[1118]; P{GD11266}v26472	VDRC	no effect	0	no effect	0	no effect	0
CG4612	CG4612	v52497	w[1118]; P{GD11372}v52497	VDRC	enhancement	-1	enhancement	-1	an effect	-1
CG4806	CG4887	v26633	w[1118]; P{GD11434}v26633	VDRC	Not done		no effect	0	no effect	0
CG4887	CG4887	v21969	w[1118]; P{GD11458}v21969	VDRC	no effect	0	no effect	0	no effect	0
CG4896	CG4896	v26652	w[1118]; P{GD11461}v26652	VDRC	no effect	0	no effect	0	no effect	0
CG5808	CG5808	v22199	w[1118]; P{GD11774}v22199	VDRC	no effect	0	no effect	0	no effect	0
CG6937	CG6937	41824	y[1] v[1]; P{y[+t7.7] v[+t1.8]=TRiP.GL01252}attP2/TM3, Sb[1]	Bloomington	no effect	0	no effect	0	no effect	0
CG7185	CG7185	34804	y[1] sc[*] v[1]; P{y[+t7.7] v[+t1.8]=TRiP.HMS00113}attP2	Bloomington	enhancement (lethal)	-3	enhancement	-3	an effect	-1
CG7804	CG7804	42579	y[1] sc[*] v[1]; P{y[+t7.7] v[+t1.8]=TRiP.HMS01884}attP2	Bloomington	no effect	0	no effect	0	no effect	0
CG7879	CG8205	v15260	w[1118]; P{GD5974}v15260	VDRC	no effect	0	no effect	0	no effect	0
CG8368	CG8368	42635	y[1] sc[*] v[1]; P{y[+t7.7] v[+t1.8]=TRiP.HMS02471}attP40	Bloomington	enhancement	-1	suppression	2	no effect	0
CG9107	CG9107	43547	y[1] sc[*] v[1]; P{y[+t7.7] v[+t1.8]=TRiP.HMS02555}attP40	Bloomington	no effect	0	no effect	0	no effect	0
CG9346	CG9346	v27013	w[1118]; P{GD14194}v27013	VDRC	no effect	0	no effect	0	no effect	0
Cnot4	CG31716	42513	y[1] v[1]; P{y[+t7.7] v[+t1.8]=TRiP.HMJ02078}attP40	Bloomington	no effect	0	no effect	0	no effect	0
cpo	CG43738	28360	y[1] v[1]; P{y[+t7.7] v[+t1.8]=TRiP.JF02996}attP2	Bloomington	no effect	0	no effect	0	no effect	0
CstF-64	CG7697	v21045	w[1118]; P{GD9942}v21045/CyO	VDRC	no effect	0	no effect	0	no effect	0
cyp33	CG4886	35611	y[1] sc[*] v[1]; P{y[+t7.7] v[+t1.8]=TRiP.GL00451}attP2/TM3, Sb[1]	Bloomington	no effect	0	no effect	0	no effect	0
eIF3-S4	CG10881	v35495	w[1118]; P{GD12611}v35495	VDRC	enhancement (lethal)	-3	suppression	3	an effect	1
eIF4B	CG10837	57305	y[1] sc[*] v[1]; P{y[+t7.7] v[+t1.8]=TRiP.HMS04503}attP40	Bloomington	no effect	0	suppression	3	no effect	0
elav	CG4262	28371	y[1] v[1]; P{y[+t7.7] v[+t1.8]=TRiP.JF03008}attP2/TM3, Sb[1]	Bloomington	no effect	0	no effect	0	no effect	0
fne	CG4396	28784	y[1] v[1]; P{y[+t7.7] v[+t1.8]=TRiP.JF03212}attP2	Bloomington	no effect	0	no effect	0	no effect	0

fus	CG8205	v107575	P{KK108164}VIE-260B	VDRC	no effect	0	no effect	0	no effect	0
glo	CG6946	33668	y[1] sc[*] v[1]; P{y[+t7.7] v[+t1.8]=TRiP.HMS00079}attP2	Bloomington	no effect	0	no effect	0	no effect	0
gw	CG31992	34796	y[1] sc[*] v[1]; P{y[+t7.7] v[+t1.8]=TRiP.HMS00105}attP2/TM3, Sb[1]	Bloomington	enhancement	-3	enhancement	-1	an effect	1
heph	CG31000	27040	y[1] v[1]; P{y[+t7.7] v[+t1.8]=TRiP.JF02366}attP2	Bloomington	no effect	0	no effect	0	no effect	0
Hrb27C	CG10377	33716	y[1] sc[*] v[1]; P{y[+t7.7] v[+t1.8]=TRiP.HMS00597}attP2	Bloomington	suppression	1	enhancement	-1	no effect	0
Hrb87F	CG12749	31244	y[1] v[1]; P{y[+t7.7] v[+t1.8]=TRiP.JF01757}attP2	Bloomington	enhancement	-2	suppression	1	no effect	0
Hrb98DE	CG9983	32351	y[1] sc[*] v[1]; P{y[+t7.7] v[+t1.8]=TRiP.HMS00342}attP2	Bloomington	enhancement	-1	enhancement	-1	no effect	0
Imp	CG1691	34977	y[1] sc[*] v[1]; P{y[+t7.7] v[+t1.8]=TRiP.HMS01168}attP2	Bloomington	no effect	0	no effect	0	no effect	0
La	CG10922	42789	y[1] v[1]; P{y[+t7.7] v[+t1.8]=TRiP.GL01159}attP2/TM3, Sb[1]	Bloomington	no effect	0	no effect	0	no effect	0
lark	CG8597	27703	y[1] v[1]; P{y[+t7.7] v[+t1.8]=TRiP.JF02783}attP2	Bloomington	no effect	0	no effect	0	no effect	0
lost	CG14648	38931	y[1] sc[*] v[1]; P{y[+t7.7] v[+t1.8]=TRiP.GL01090}attP2	Bloomington	no effect	0	no effect	0	no effect	0
LS2	CG3162	55154	y[1] sc[*] v[1]; P{y[+t7.7] v[+t1.8]=TRiP.HMC03812}attP40	Bloomington	no effect	0	no effect	0	no effect	0
musashi	CG5099	4160	msi[1]/TM3, Sb[1]	Bloomington	enhancement	-2	enhancement	-1	no effect	0
Nelf-E	CG5994	32835	y[1] sc[*] v[1]; P{y[+t7.7] v[+t1.8]=TRiP.HMS00525}attP2/TM3, Sb[1]	Bloomington	no effect	0	no effect	0	no effect	0
nito	CG2910	34848	y[1] sc[*] v[1]; P{y[+t7.7] v[+t1.8]=TRiP.HMS00166}attP2	Bloomington	no effect	0	no effect	0	no effect	0
nonA	CG4211	52933	y[1] sc[*] v[1]; P{y[+t7.7] v[+t1.8]=TRiP.HMC03675}attP40	Bloomington	no effect	0	no effect	0	no effect	0
nonA-like	CG10328	52934	y[1] sc[*] v[1]; P{y[+t7.7] v[+t1.8]=TRiP.HMC03676}attP40	Bloomington	no effect	0	no effect	0	no effect	0
orb2	CG43782	27050	y[1] v[1]; P{y[+t7.7] v[+t1.8]=TRiP.JF02376}attP2	Bloomington	no effect	0	no effect	0	no effect	0
pAbp	CG5119	36127	y[1] sc[*] v[1]; P{y[+t7.7] v[+t1.8]=TRiP.HMS01542}attP40	Bloomington	no effect	0	no effect	0	no effect	0
pAbp2	CG2163	34602	y[1] sc[*] v[1]; P{y[+t7.7] v[+t1.8]=TRiP.HMS00553}attP2	Bloomington	enhancement	-2	no effect	0	no effect	0
pUf68	CG12085	34785	y[1] sc[*] v[1]; P{y[+t7.7] v[+t1.8]=TRiP.HMS00094}attP2	Bloomington	enhancement	-2	enhancement	-2	no effect	0
Rbp1	CG17136	v21083	w[1118]; P{GD9289}v21083/TM3	VDRC	no effect	0	suppression	2	no effect	0
Rbp1L	CG1987	44100	y[1] sc[*] v[1]; P{y[+t7.7] v[+t1.8]=TRiP.HMS02820}attP40	Bloomington	no effect	0	suppression	3	no effect	0
Rbp2	CG4429	43275	y[1] sc[*] v[1]; P{y[+t7.7] v[+t1.8]=TRiP.GLC01464}attP2	Bloomington	no effect	0	no effect	0	no effect	0
Rbp6	CG32169	v29799	w[1118]; P{GD15236}v29799/CyO	VDRC	enhancement	-1	no effect	0	no effect	0
Rbp9	CG3151	28669	y[1] v[1]; P{y[+t7.7] v[+t1.8]=TRiP.JF03084}attP2	Bloomington	no effect	0	no effect	0	no effect	0
Ref1	CG1101	34626	y[1] sc[*] v[1]; P{y[+t7.7] v[+t1.8]=TRiP.HMS01301}attP2/TM3, Sb[1]	Bloomington	suppression	2	suppression	3	no effect	0
Ref2	CG17031	v32829	w[1118]; P{GD9267}v32829	VDRC	no effect	1	no effect	1	no effect	0
rin	CG9412	33392	y[1] sc[*] v[1]; P{y[+t7.7] v[+t1.8]=TRiP.HMS00269}attP2/TM3, Sb[1]	Bloomington	no effect	0	no effect	0	no effect	0
Rnp4F	CG3312	35457	y[1] sc[*] v[1]; P{y[+t7.7] v[+t1.8]=TRiP.GL00383}attP2	Bloomington	no effect	0	no effect	0	no effect	0
RnpS1	CG16788	36580	y[1] sc[*] v[1]; P{y[+t7.7] v[+t1.8]=TRiP.GL00540}attP2	Bloomington	no effect	0	no effect	-1	no effect	0
Rox8	CG5422	32472	y[1] sc[*] v[1]; P{y[+t7.7] v[+t1.8]=TRiP.HMS00472}attP2	Bloomington	no effect	0	no effect	0	no effect	0
rump	CG9373	42665	y[1] sc[*] v[1]; P{y[+t7.7] v[+t1.8]=TRiP.HMS02501}attP40	Bloomington	no effect	0	no effect	0	no effect	0
Saf-B	CG6997	51759	y[1] v[1]; P{y[+t7.7] v[+t1.8]=TRiP.HMC03311}attP2	Bloomington	no effect/black patches?	0	suppression	1	no effect	0
SC35	CG5442	v40590	w[1118]; P{GD11654}v40590	VDRC	no effect	0	no effect	0	no effect	0
Set1	CG40351	40931	y[1] sc[*] v[1]; P{y[+t7.7] v[+t1.8]=TRiP.HMS02179}attP40	Bloomington	no effect	0	no effect	0	no effect	0
SF2	CG6987	32367	y[1] sc[*] v[1]; P{y[+t7.7] v[+t1.8]=TRiP.HMS00358}attP2	Bloomington	no effect	0	suppression	2	no effect	0
shep	CG32423	43545	y[1] sc[*] v[1]; P{y[+t7.7] v[+t1.8]=TRiP.HMS02666}attP40	Bloomington	enhancement	-3	suppression	3	no effect	0
sm	CG9218	v28117	w[1118]; P{GD12545}v28117/TM3	VDRC	no effect	0	no effect	0	no effect	0
snf	CG4528	34953	y[1] sc[*] v[1]; P{y[+t7.7] v[+t1.8]=TRiP.HMS01067}attP2/TM3, Sb[1]	Bloomington	no effect	0	no effect	0	no effect	0
Snp	CG44248	33434	y[1] sc[*] v[1]; P{y[+t7.7] v[+t1.8]=TRiP.HMS00321}attP2	Bloomington	no effect	0	no effect	0	no effect	0
snRNP-U1-70K	CG8749	33396	y[1] sc[*] v[1]; P{y[+t7.7] v[+t1.8]=TRiP.HMS00274}attP2	Bloomington	enhancement	-3	enhancement	-2	lethal	-3
Spargel	CG9809	33915	y[1] sc[*] v[1]; P{y[+t7.7] v[+t1.8]=TRiP.HMS00858}attP2	Bloomington	no effect	0	no effect	0	no effect	0
spen	CG18497	33398	y[1] sc[*] v[1]; P{y[+t7.7] v[+t1.8]=TRiP.HMS00276}attP2	Bloomington	enhancement	-3	enhancement	-3	an effect	-3
Spf45	CG17540	41954	y[1] sc[*] v[1]; P{y[+t7.7] v[+t1.8]=TRiP.HMS02351}attP2	Bloomington	no effect	0	no effect	0	no effect	0
Spx	CG3780	v40471	w[1118]; P{GD11072}v40471	VDRC	enhancement	-3	enhancement	-2	no effect	0
sqd	CG16901	31302	y[1] v[1]; P{y[+t7.7] v[+t1.8]=TRiP.JF01248}attP2	Bloomington	no effect	0	no effect	0	no effect	0
Srp54	CG6340	30533	y[1] sc[*] v[1]; P{y[+t7.7] v[+t1.8]=TRiP.HM05224}attP2	Bloomington	no effect	0	no effect	0	no effect	0
swm	CG10084	52935	y[1] sc[*] v[1]; P{y[+t7.7] v[+t1.8]=TRiP.HMC03677}attP40	Bloomington	suppression	1	suppression	3	no effect	0
Sxl	CG43770	34393	y[1] sc[*] v[1]; P{y[+t7.7] v[+t1.8]=TRiP.HMS00609}attP2	Bloomington	no effect	0	no effect	0	no effect	0
Syp	CG17838	v33011	w[1118]; P{GD9477}v33011	VDRC	no effect	0	no effect	0	no effect	0
TBPH	CG10327	39014	y[1] v[1]; P{y[+t7.7] v[+t1.8]=TRiP.HMS01932}attP40	Bloomington	no effect	0	no effect	0	no effect	0

tra2	CG10128	28018	y[1] v[1]; P{y[+t7.7] v[+t1.8]=TRiP.JF02852}attP2	Bloomington	no effect	0	no effect	0	no effect	0
tsu	CG8781	28955	y[1] v[1]; P{y[+t7.7] v[+t1.8]=TRiP.HM05166}attP2	Bloomington	no effect	0	suppression	3	no effect	0
U2af38	CG3582	29304	y[1] v[1]; P{y[+t7.7] v[+t1.8]=TRiP.JF02444}attP2	Bloomington	no effect	0	suppression	3	no effect	0
U2af50	CG9998	27542	y[1] v[1]; P{y[+t7.7] v[+t1.8]=TRiP.JF02693}attP2	Bloomington	no effect	0	no effect	0	no effect	0
x16	CG10203	51468	y[1] v[1]; P{y[+t7.7] v[+t1.8]=TRiP.HMC03209}attP2	Bloomington	no effect	0	no effect	0	no effect	0
