# Supplementary material for: Drosophila Ref1/ALYREF regulates transcription and toxicity associated with ALS/FTD disease etiologies
Source: Acta Neuropathol Commun. 2019 Apr 29;7:65. doi: 10.1186/s40478-019-0710-x (PMC6487524; doi:10.1186/s40478-019-0710-x)
Supplement: Supplementary file 2 — Figure S1. Extended data characterizing Ref1 RNAi. Figure S2. Uncropped western blot images. (DOCX 977 kb) [file 40478_2019_710_MOESM2_ESM.docx]

**Targeting *ALYREF* suppresses neurodegeneration in TDP-43 associated ALS/FTD.**

Amit Berson^1,*^, Lindsey D. Goodman^2,*^, Ashley Sartoris^1^, Charlton G. Otte^1^, James Aykit^1^, Virginia M.-Y. Lee ^3^, John Q. Trojanowski^3^ , and Nancy M. Bonini^1,**^

# **Figure S1**


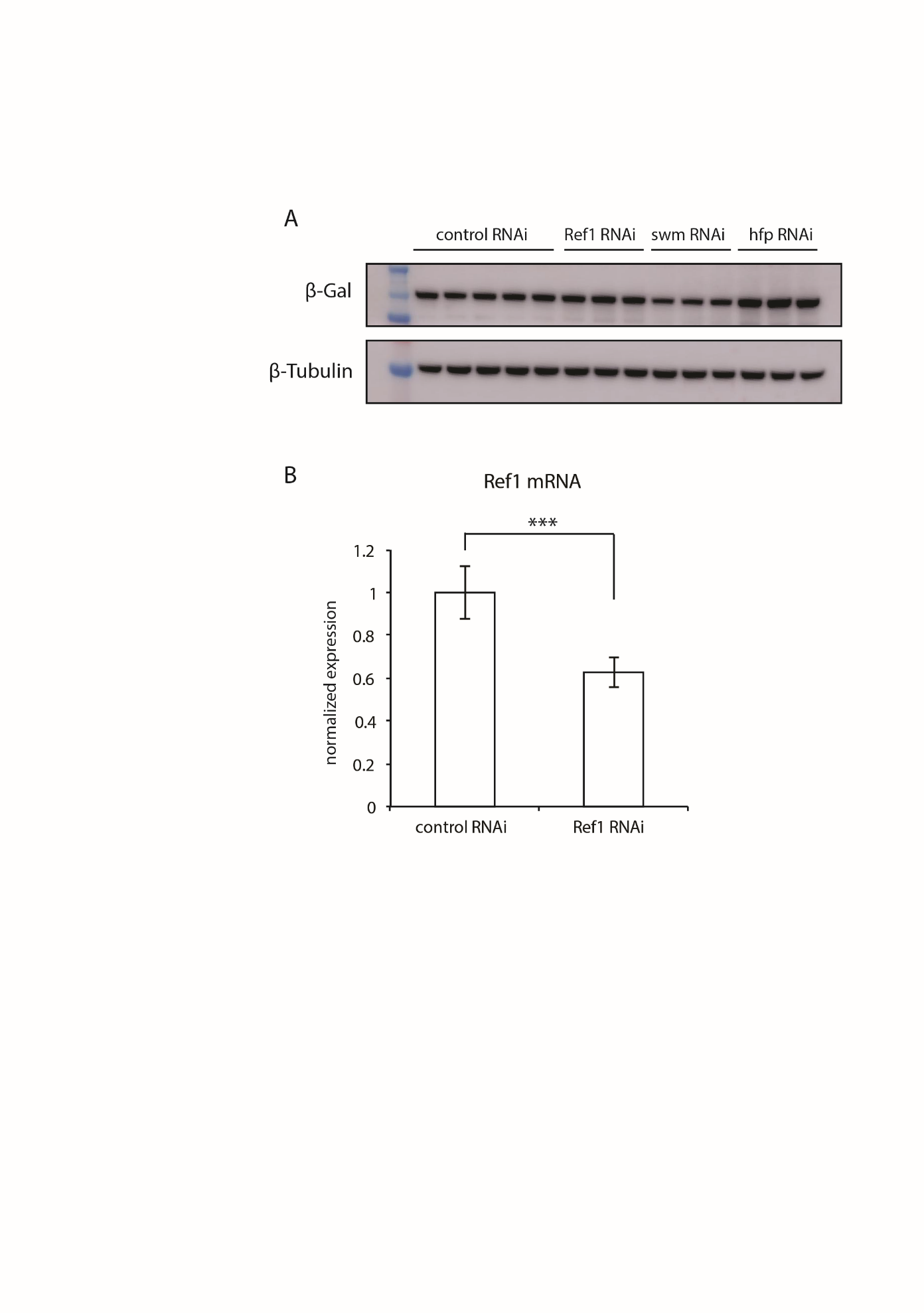


**Figure S1: Extended data characterizing *Ref1* RNAi.**

**A.** Control, Ref, Swm, or HFP RNAi were expressed in control *LacZ* animals using Gmr-GAL4 to express transgenes. Western blot for the β-galactosidase protein revealed effects on control *LacZ* expression due to the RNAi. **B.** qRT-PCR data showing knockdown efficiency of Ref1 RNAi relative to control RNAi in adult flies using Daughterless-Gal4[geneswitch] to express transgenes.

# **Figure S2**

**
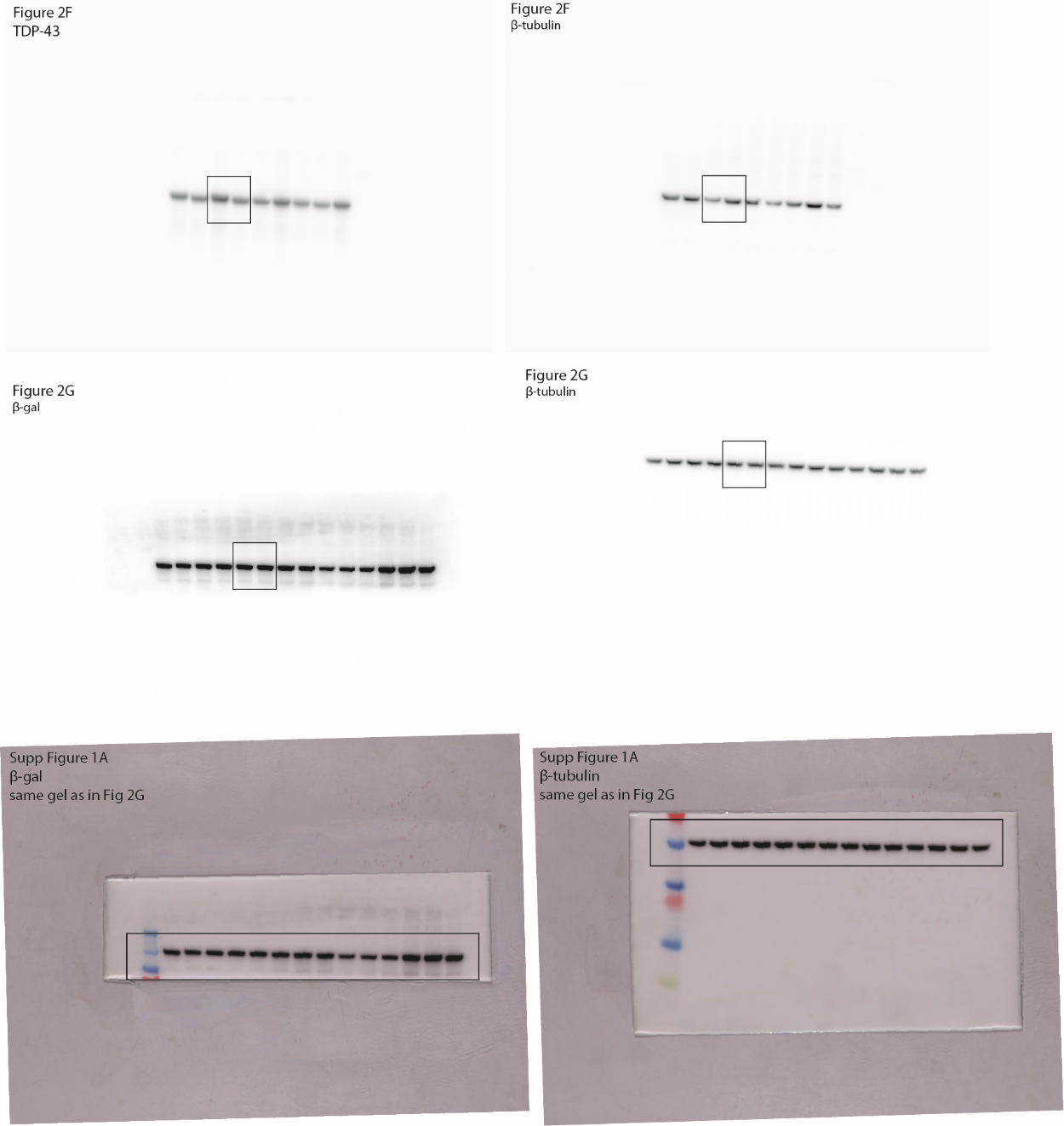
**

**Figure S2: Uncropped western blot images.**
