# Supplementary material for: Drosophila Ref1/ALYREF regulates transcription and toxicity associated with ALS/FTD disease etiologies
Source: Acta Neuropathol Commun. 2019 Apr 29;7:65. doi: 10.1186/s40478-019-0710-x (PMC6487524; doi:10.1186/s40478-019-0710-x)
Supplement: Supplementary file 3 — Table S2. Patient information. (PDF 56 kb) [file 40478_2019_710_MOESM3_ESM.pdf]

Table S2

ID	clinical diagnosis	C9ORF72	Sex	Age-at-Death
1	Normal		Male	42
2	Normal		Male	55
3	Normal		Male	57
4	Normal		Male	59
5	Normal		Male	62
6	Normal		Male	67
7	Normal		Male	67
8	Normal		Male	68
9	Normal		Male	70
10	ALS		Male	57
11	ALS		Male	58
12	ALS	Expansion	Male	58
13	ALS	Expansion	Male	60
14	ALS		Male	67
15	ALS	Expansion	Male	70
