# Supplementary material for: Drosophila Ref1/ALYREF regulates transcription and toxicity associated with ALS/FTD disease etiologies
Source: Acta Neuropathol Commun. 2019 Apr 29;7:65. doi: 10.1186/s40478-019-0710-x (PMC6487524; doi:10.1186/s40478-019-0710-x)
Supplement: Supplementary file 4 — Table S3. Drosophila RNAi lines. (PDF 98 kb) [file 40478_2019_710_MOESM4_ESM.pdf]

Table S3

Gene	Stock #	Genotype	Source
A2bp1	32476	y[1] sc[*] v[1]; P{y[+t7.7] v[+t1.8]=TRiP.HMS00478}attP2	Bloomington
aret	38983	y[1] v[1]; P{y[+t7.7] v[+t1.8]=TRiP.HMS01899}attP40	Bloomington
barc	42504	y[1] v[1]; P{y[+t7.7] v[+t1.8]=TRiP.HMJ02069}attP40	Bloomington
bol	21536	w[1118]; P{GD10525}v21536	VDRC
bru-2	50631	y[1] sc[*] v[1]; P{y[+t7.7] v[+t1.8]=TRiP.HMC02998}attP2	Bloomington
bru-3	43318	y[1] sc[*] v[1]; P{y[+t7.7] v[+t1.8]=TRiP.HMS02702}attP40	Bloomington
B52	37519	y[1] sc[*] v[1]; P{y[+t7.7] v[+t1.8]=TRiP.HMS01661}attP40	Bloomington
caz	34839	y[1] sc[*] v[1]; P{y[+t7.7] v[+t1.8]=TRiP.HMS00156}attP2	Bloomington
Cbp20	42596	y[1] sc[*] v[1]; P{y[+t7.7] v[+t1.8]=TRiP.HMS02428}attP40	Bloomington
Cnot4	42513	y[1] v[1]; P{y[+t7.7] v[+t1.8]=TRiP.HMJ02078}attP40	Bloomington
cpo	28360	y[1] v[1]; P{y[+t7.7] v[+t1.8]=TRiP.JF02996}attP2	Bloomington
CstF-64	31812	w[1118]; P{w[+mC]=EP}CstF-64[G16431]	Bloomington
CstF-64	21045	w[1118]; P{GD9942}v21045/CyO	VDRC
cyp33	35611	y[1] sc[*] v[1]; P{y[+t7.7] v[+t1.8]=TRiP.GL00451}attP2/TM3, Sb[1]	Bloomington
elav	28371	y[1] v[1]; P{y[+t7.7] v[+t1.8]=TRiP.JF03008}attP2/TM3, Sb[1]	Bloomington
eIF3-S4	35495	w[1118]; P{GD12611}v35495	VDRC
eIF-4B	57305	y[1] sc[*] v[1]; P{y[+t7.7] v[+t1.8]=TRiP.HMS04503}attP40	Bloomington
fne	28784	y[1] v[1]; P{y[+t7.7] v[+t1.8]=TRiP.JF03212}attP2	Bloomington
fus	107575	P{KK108164}VIE-260B	VDRC
glo	33668	y[1] sc[*] v[1]; P{y[+t7.7] v[+t1.8]=TRiP.HMS00079}attP2	Bloomington
gw	34796	y[1] sc[*] v[1]; P{y[+t7.7] v[+t1.8]=TRiP.HMS00105}attP2/TM3, Sb[1]	Bloomington
heph	27040	y[1] v[1]; P{y[+t7.7] v[+t1.8]=TRiP.JF02366}attP2	Bloomington
Hrb27C	33716	y[1] sc[*] v[1]; P{y[+t7.7] v[+t1.8]=TRiP.HMS00597}attP2	Bloomington
Hrb87F	31244	y[1] v[1]; P{y[+t7.7] v[+t1.8]=TRiP.JF01757}attP2	Bloomington
Hrb98DE	32351	y[1] sc[*] v[1]; P{y[+t7.7] v[+t1.8]=TRiP.HMS00342}attP2	Bloomington
Imp	34977	y[1] sc[*] v[1]; P{y[+t7.7] v[+t1.8]=TRiP.HMS01168}attP2	Bloomington
La	42789	y[1] v[1]; P{y[+t7.7] v[+t1.8]=TRiP.GL01159}attP2/TM3, Sb[1]	Bloomington
lark	27703	y[1] v[1]; P{y[+t7.7] v[+t1.8]=TRiP.JF02783}attP2	Bloomington
lost	38931	y[1] sc[*] v[1]; P{y[+t7.7] v[+t1.8]=TRiP.GL01090}attP2	Bloomington
LS2	55154	y[1] sc[*] v[1]; P{y[+t7.7] v[+t1.8]=TRiP.HMC03812}attP40	Bloomington
musashi[1]	4160	msi[1]/TM3, Sb[1]	Bloomington
musashi[2]	4161	msi[2]/TM3, Sb[1]	Bloomington
Nelf-E	32835	y[1] sc[*] v[1]; P{y[+t7.7] v[+t1.8]=TRiP.HMS00525}attP2/TM3, Sb[1]	Bloomington
nito	34848	y[1] sc[*] v[1]; P{y[+t7.7] v[+t1.8]=TRiP.HMS00166}attP2	Bloomington
nonA	52933	y[1] sc[*] v[1]; P{y[+t7.7] v[+t1.8]=TRiP.HMC03675}attP40	Bloomington
nonA-like	52934	y[1] sc[*] v[1]; P{y[+t7.7] v[+t1.8]=TRiP.HMC03676}attP40	Bloomington
orb2	27050	y[1] v[1]; P{y[+t7.7] v[+t1.8]=TRiP.JF02376}attP2	Bloomington
pAbp	36127	y[1] sc[*] v[1]; P{y[+t7.7] v[+t1.8]=TRiP.HMS01542}attP40	Bloomington
pAbp2	34602	y[1] sc[*] v[1]; P{y[+t7.7] v[+t1.8]=TRiP.HMS00553}attP2	Bloomington
pUf68	34785	y[1] sc[*] v[1]; P{y[+t7.7] v[+t1.8]=TRiP.HMS00094}attP2	Bloomington
Rbp1	21083	w[1118]; P{GD9289}v21083/TM3	VDRC
Rbp1-like	44100	y[1] sc[*] v[1]; P{y[+t7.7] v[+t1.8]=TRiP.HMS02820}attP40	Bloomington
Rbp2	43275	y[1] sc[*] v[1]; P{y[+t7.7] v[+t1.8]=TRiP.GLC01464}attP2	Bloomington
Rbp6	29799	w[1118]; P{GD15236}v29799/CyO	VDRC
Rbp9	28669	y[1] v[1]; P{y[+t7.7] v[+t1.8]=TRiP.JF03084}attP2	Bloomington
Ref1	34626	y[1] sc[*] v[1]; P{y[+t7.7] v[+t1.8]=TRiP.HMS01301}attP2/TM3, Sb[1]	Bloomington
Ref2	32829	w[1118]; P{GD9267}v32829	VDRC
rin	33392	y[1] sc[*] v[1]; P{y[+t7.7] v[+t1.8]=TRiP.HMS00269}attP2/TM3, Sb[1]	Bloomington
RnpS1	36580	y[1] sc[*] v[1]; P{y[+t7.7] v[+t1.8]=TRiP.GL00540}attP2	Bloomington
Rnp4F	35457	y[1] sc[*] v[1]; P{y[+t7.7] v[+t1.8]=TRiP.GL00383}attP2	Bloomington
Rox8	32472	y[1] sc[*] v[1]; P{y[+t7.7] v[+t1.8]=TRiP.HMS00472}attP2	Bloomington
rump	42665	y[1] sc[*] v[1]; P{y[+t7.7] v[+t1.8]=TRiP.HMS02501}attP40	Bloomington
Saf-B	51759	y[1] v[1]; P{y[+t7.7] v[+t1.8]=TRiP.HMC03311}attP2	Bloomington
SC35	40590	w[1118]; P{GD11654}v40590	VDRC
Set1	40931	y[1] sc[*] v[1]; P{y[+t7.7] v[+t1.8]=TRiP.HMS02179}attP40	Bloomington
SF2	32367	y[1] sc[*] v[1]; P{y[+t7.7] v[+t1.8]=TRiP.HMS00358}attP2	Bloomington
shep	43545	y[1] sc[*] v[1]; P{y[+t7.7] v[+t1.8]=TRiP.HMS02666}attP40	Bloomington
sm	28117	w[1118]; P{GD12545}v28117/TM3	VDRC
snf	34953	y[1] sc[*] v[1]; P{y[+t7.7] v[+t1.8]=TRiP.HMS01067}attP2/TM3, Sb[1]	Bloomington
Snp	33434	y[1] sc[*] v[1]; P{y[+t7.7] v[+t1.8]=TRiP.HMS00321}attP2	Bloomington
snRNP-U1-70K	33396	y[1] sc[*] v[1]; P{y[+t7.7] v[+t1.8]=TRiP.HMS00274}attP2	Bloomington

Spargel	33915	y[1] sc[*] v[1]; P{y[+t7.7] v[+t1.8]=TRiP.HMS00858}attP2	Bloomington
spen	33398	y[1] sc[*] v[1]; P{y[+t7.7] v[+t1.8]=TRiP.HMS00276}attP2	Bloomington
Spf45	41954	y[1] sc[*] v[1]; P{y[+t7.7] v[+t1.8]=TRiP.HMS02351}attP2	Bloomington
Spx	40471	w[1118]; P{GD11072}v40471	VDRC
sqd	31302	y[1] v[1]; P{y[+t7.7] v[+t1.8]=TRiP.JF01248}attP2	Bloomington
Srp54	30533	y[1] sc[*] v[1]; P{y[+t7.7] v[+t1.8]=TRiP.HM05224}attP2	Bloomington
swm	28548	y[1] v[1]; P{y[+t7.7] v[+t1.8]=TRiP.HM05034}attP2	Bloomington
swm	52935	y[1] sc[*] v[1]; P{y[+t7.7] v[+t1.8]=TRiP.HMC03677}attP40	Bloomington
Sxl	34393	y[1] sc[*] v[1]; P{y[+t7.7] v[+t1.8]=TRiP.HMS00609}attP2	Bloomington
Syp	33011	w[1118]; P{GD9477}v33011	VDRC
TBPH	39014	y[1] v[1]; P{y[+t7.7] v[+t1.8]=TRiP.HMS01932}attP40	Bloomington
tra2	28018	y[1] v[1]; P{y[+t7.7] v[+t1.8]=TRiP.JF02852}attP2	Bloomington
tsu	28955	y[1] v[1]; P{y[+t7.7] v[+t1.8]=TRiP.HM05166}attP2	Bloomington
U2af38	29304	y[1] v[1]; P{y[+t7.7] v[+t1.8]=TRiP.JF02444}attP2	Bloomington
U2af50	27542	y[1] v[1]; P{y[+t7.7] v[+t1.8]=TRiP.JF02693}attP2	Bloomington
x16	51468	y[1] v[1]; P{y[+t7.7] v[+t1.8]=TRiP.HMC03209}attP2	Bloomington
CG1316	23851	w[1118]; P{GD7942}v23851/TM3	VDRC
CG2931	20946	w[1118]; P{GD9858}v20946	VDRC
CG3294	27296	y[1] v[1]; P{y[+t7.7] v[+t1.8]=TRiP.JF02606}attP2	Bloomington
CG3335	17737	w[1118]; PBac{w[+mC]=PB}CG3335[c05958]/TM6B, Tb[1]	Bloomington
CG4119	26395	w[1118]; P{GD11222}v26395	VDRC
CG4266	26472	w[1118]; P{GD11266}v26472	VDRC
CG4612	52497	w[1118]; P{GD11372}v52497	VDRC
CG4806	26633	w[1118]; P{GD11434}v26633	VDRC
CG4887	21969	w[1118]; P{GD11458}v21969	VDRC
CG4896	26652	w[1118]; P{GD11461}v26652	VDRC
CG5808	22199	w[1118]; P{GD11774}v22199	VDRC
CG6937	41824	y[1] v[1]; P{y[+t7.7] v[+t1.8]=TRiP.GL01252}attP2/TM3, Sb[1]	Bloomington
CG7185 (wah)	34804	y[1] sc[*] v[1]; P{y[+t7.7] v[+t1.8]=TRiP.HMS00113}attP2	Bloomington
CG7804	42579	y[1] sc[*] v[1]; P{y[+t7.7] v[+t1.8]=TRiP.HMS01884}attP2	Bloomington
CG7879	15260	w[1118]; P{GD5974}v15260	VDRC
CG8368	42635	y[1] sc[*] v[1]; P{y[+t7.7] v[+t1.8]=TRiP.HMS02471}attP40	Bloomington
CG9107	43547	y[1] sc[*] v[1]; P{y[+t7.7] v[+t1.8]=TRiP.HMS02555}attP40	Bloomington
CG9346	27013	w[1118]; P{GD14194}v27013	VDRC
CG10466	55263	y[1] sc[*] v[1]; P{y[+t7.7] v[+t1.8]=TRiP.HMC03950}attP40	Bloomington
CG10948	31388	w[1118]; P{GD7139}v31388	VDRC
CG10948	55280	y[1] sc[*] v[1]; P{y[+t7.7] v[+t1.8]=TRiP.HMC03967}attP40	Bloomington
CG11266	44431	y[1] sc[*] v[1]; P{y[+t7.7] v[+t1.8]=TRiP.GLC01382}attP2/TM3, Sb[1]	Bloomington
CG11454	not available	GLC01372	TRiP center
CG12288	56037	y[1] sc[*] v[1]; P{y[+t7.7] v[+t1.8]=TRiP.HMC04345}attP40	Bloomington
CG13298	42873	y[1] sc[*] v[1]; P{y[+t7.7] v[+t1.8]=TRiP.HMS02566}attP40	Bloomington
CG14641	38790	w[1118]; P{GD8388}v38790/TM3	VDRC
CG14718	38331	y[1] sc[*] v[1]; P{y[+t7.7] v[+t1.8]=TRiP.HMS01798}attP2	Bloomington
CG15440	57807	y[1] v[1]; P{y[+t7.7] v[+t1.8]=TRiP.HMJ21815}attP40	Bloomington
CG17187	40051	w[1118]; P{GD9309}v40051	VDRC
CG17187	55702	y[1] sc[*] v[1]; P{y[+t7.7] v[+t1.8]=TRiP.HMC03923}attP40	Bloomington
CG18259	34081	y[1] sc[*] v[1]; P{y[+t7.7] v[+t1.8]=TRiP.HMS01089}attP2/TM3, Sb[1]	Bloomington
CG33713/CG33714	55374	y[1] sc[*] v[1]; P{y[+t7.7] v[+t1.8]=TRiP.HMC04062}attP40/CyO	Bloomington
CG34354	33674	y[1] sc[*] v[1]; P{y[+t7.7] v[+t1.8]=TRiP.HMS00538}attP2	Bloomington
CG34362	39749	w[1118]; P{GD7852}v39749	VDRC
CG42458	42506	y[1] v[1]; P{y[+t7.7] v[+t1.8]=TRiP.HMJ02071}attP40	Bloomington
