# Supplementary material for: Drosophila Ref1/ALYREF regulates transcription and toxicity associated with ALS/FTD disease etiologies
Source: Acta Neuropathol Commun. 2019 Apr 29;7:65. doi: 10.1186/s40478-019-0710-x (PMC6487524; doi:10.1186/s40478-019-0710-x)
Supplement: Supplementary file 5 — Table S4. Primer sequences. (PDF 57 kb) [file 40478_2019_710_MOESM5_ESM.pdf]

Table S4

primer name	sequence 5'-3'	Reference
TDP-43-F	GCTGGGGAAATCTGGTGTAT	This manuscript
TDP-43-R	CGGATGTTTTCTGGACTGCT	This manuscript
UAS-G4C2-F	AGACTCGGTACCGGATCCTC	Goodman et al., Nat Neuro, in press
UAS-G4C2-R	GCTCCCATTCATCAGTTCCA	Goodman et al., Nat Neuro, in press
UAS-LDS-G4C2-F	ACGTAAACGGCCACAAGTTC	Goodman et al., Nat Neuro, in press
UAS-LDS-G4C2-R	AAGTCGTGCTGCTTCATGTG	Goodman et al., Nat Neuro, in press
Tau#1-F	AAGGTGACCTCCAAGTGTGG	This manuscript
Tau#1-R	GGACGTGGGTGATATTGTCC	This manuscript
beta Gal_#2-F	GTGCGGATTGAAAATGGTCT	This manuscript
beta Gal_#2-R	GACCTGACCATGCAGAGGAT	This manuscript
Ref1-F	CCGTTCAGAAGGCCAAGTT	This manuscript
Ref1-R	AGTCCAGGTTACCGACGATG	This manuscript
RpS20-F	CCGCATCACCCTGACATCC	Colinet et al., FEBS J. 2010 Jan;277(1):174-85
RpS20-R	TGGTGATGCGAAGGGTCTTG	Colinet et al., FEBS J. 2010 Jan;277(1):174-85
alpha Tubulin-F	CACACCACCCTGGAGCATTC	https://www.flyrnai.org/flyprimerbank
alpha Tubulin-R	CCAATCAGACGGTTCAGGTTG	https://www.flyrnai.org/flyprimerbank
